# Exploring the Mechanism of Sempervirine Inhibiting Glioblastoma Invasion Based on Network Pharmacology and Bioinformatics

**DOI:** 10.3390/ph17101318

**Published:** 2024-10-02

**Authors:** Bingqiang Zhang, Wenyi Wang, Yu Song, Huixian Chen, Xinxin Lin, Jingjing Chen, Ying Chen, Jinfang Huang, Desen Li, Shuisheng Wu

**Affiliations:** 1 College of Pharmacy, Fujian University of Traditional Chinese Medicine, Fuzhou 350122, China; 2220408001@fjtcm.edu.cn (B.Z.); 2000015@fjtcm.edu.cn (Y.S.); 13599571768@163.com (H.C.); 2220456034@fjtcm.edu.cn (X.L.); 2220456032@fjtcm.edu.cn (J.C.); 20190941282@bucm.edu.cn (Y.C.); 2 Innovation and Transformation Center, Fujian University of Traditional Chinese Medicine, Fuzhou 350122, China; wangwy@fjtcm.edu.cn; 3 Fuzhou First General Hospital, Fuzhou 350009, China; huangjinfang@fzsdyyy.com

**Keywords:** glioblastoma, sempervirine, invasion, AKT phosphorylation, matrix metalloproteinases

## Abstract

**Background**: Invasion is an important characteristic of the malignancy of glioblastoma (GBM) and a significant prognostic factor. Sempervirine (SPV), a yohimbine-type alkaloid, has been proven to inhibit GBM cells proliferation in previous research and found to have a potential effect in anti-invasion, but its mechanism of anti-invasion is still unknown. **Methods**: To explore its pharmacodynamics in inhibiting GBM cell invasion in this study, we combined network pharmacology and bioinformatics to comprehensive exploratory analysis of SPV and verified the mechanism in vitro. **Results**: Firstly, targets of SPV and invasion-related genes were collected from public databases. Moreover, GBM samples were obtained to analyze differentially expressed genes (DEGs) from The Cancer Genome Atlas (TCGA). Then, the relevant targets of SPV inhibiting GBM invasion (SIGI) were obtained through the intersection of the three gene sets. Further, GO and KEGG analysis showed that the targets of SIGI were heavily enriched in the AKT signaling pathway. Subsequently, based on the method of machine learning, a clinical prognostic model of the relevant targets of SIGI was constructed using GBM samples from TCGA and the Gene Expression Omnibus (GEO). A four-genes model (*DUSP6*, *BMP2*, *MMP2*, and *MMP13*) was successfully constructed, and Vina Scores of MMP2 and MMP13 in molecular docking were higher, which may be the main targets of SIGI. Then, the effect of SIGI was confirmed via functional experiments on invasion, migration, and adhesion assay, and the effect involved changes in the expressions of p-AKT, MMP2 and MMP13. Finally, combined with AKT activator (SC79) and inhibitor (MK2206), we further confirmed that SPV inhibits GBM invasion through AKT phosphorylation. **Conclusions**: This study provides valuable and an expected point of view into the regulation of AKT phosphorylation and inhibition of GBM invasion by SPV.

## 1. Introduction

Glioma is the most common primary central nervous system (CNS) tumor in humans, and glioblastoma (GBM), with a high rate of invasion and mortality, is the highest-grade glioma, and often diffusely infiltrates adjacent brain tissues [1]. The clinical standard of care (SOC) remains to be tumor resection and radiation therapy (RT) with concurrent temozolomide (TMZ) for patients with newly diagnosed GBM. While this regimen has been published for over 10 years, currently the median overall survival time of GBM patients is still less than 16 months [2]. TMZ is the only first-line chemotherapy for glioma, but more than 50% of GBM patients gradually acquire TMZ resistance, and because of neural function in certain brain regions, it is impossible to eradicate infiltrating tumor cells by surgical resection, which resulted in recurrence and mortality rates approaching 100% [3,4]. Therefore, it is meaningful to clarify a mechanism of GBM invasion that can provide direction to develop relevant drugs. With the deepening study of GBM cells invasion, scholars have found that the increase in matrix metalloproteinase (MMPs) level is strongly related to the invasion of tumor cells. The ability of tumor cells to digest extracellular matrix (ECM) by secreting proteolytic enzymes is closely related to their ability to invade tissues [5,6,7]. For example, the literature has shown that c-Cbl, a multifunctional adaptor and an E3 ubiquitin ligase, promotes glioma invasion by upregulation of MMP2 [8]. Overexpression of chemokine receptor type 4 (CXCR4) has also been demonstrated to induce the expression of MMP2 to degrade ECM and promote glioma invasion and migration [9]. Similarly, it has also been shown that the inhibition of MMP2 and MMP9 by *EFEMP2* knockdown resulted in a reduction in glioma invasion [10]. These reports on glioma invasion showed a correlation between matrix metalloproteinases and glioma invasion. In most tumors, abnormal activation of the AKT signal is correlated with invasion, migration, and other malignant phenotype. Meanwhile, there are studies shown that the high expression of MMPs can be induced by the activation of the AKT signal, thus promoting the occurrence of tumor invasion and migration [11,12].

**Figure 1:**
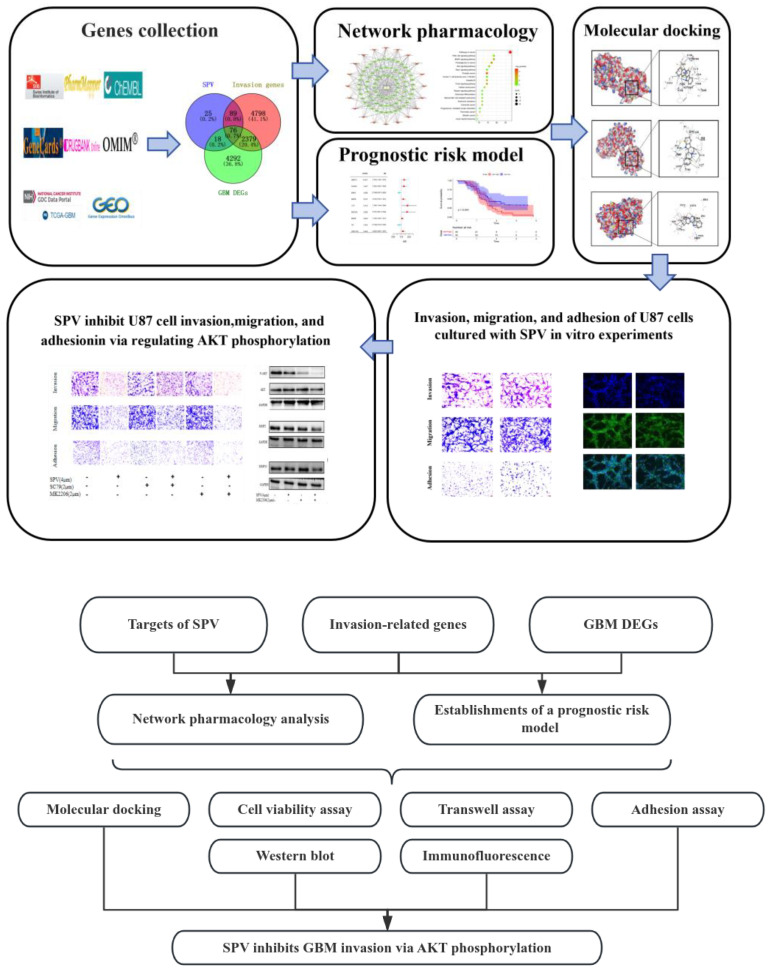
Framework on basis of experimental method to research the targets of SIGI.

Sempervirine (SPV) is a yohimbine-type alkaloid that has been proven to be effective in certain diseases of inflammation, mental, and cancer [13]. In pharmacological research, SPV has shown good value in cases of glioma, liver cancer, and testicular cancer. In human hepatocellular carcinoma, SPV has been proven to regulate the *P53* pathway to arrest the cell cycle and induce apoptosis and inhibit cell proliferation through the Wnt/β-catenin pathway [14]. In anti-testicular cancer, SPV accumulates in the nucleolus and binds to RNA to inhibit RNA synthesis in tumor cells [15]. Our previous studies have proven that activation of the AKT/mTOR pathway to induce apoptosis and autophagy is one of the mechanisms by which SPV inhibits proliferation of glioma cells [16]. However, it is not yet clear whether SPV is effective in invading glioblastoma. Based on the construction of the GBM aggressive gene prognosis model and SPV network pharmacological analysis, we found that SPV has the potential to inhibit GBM invasion, but it is unclear in the mechanism of inhibiting GBM invasion. This study further explored the mechanism of SPV inhibiting GBM invasion based on previous studies and verified it through in vitro experiments, providing lead compounds for the development of new GBM therapeutic drugs. Figure 1 is a framework based on experimental method to research the targets of SIGI.

## 2. Results

### 2.1. Identifying the Targets and Pathways of SIGI

**Figure 2:**
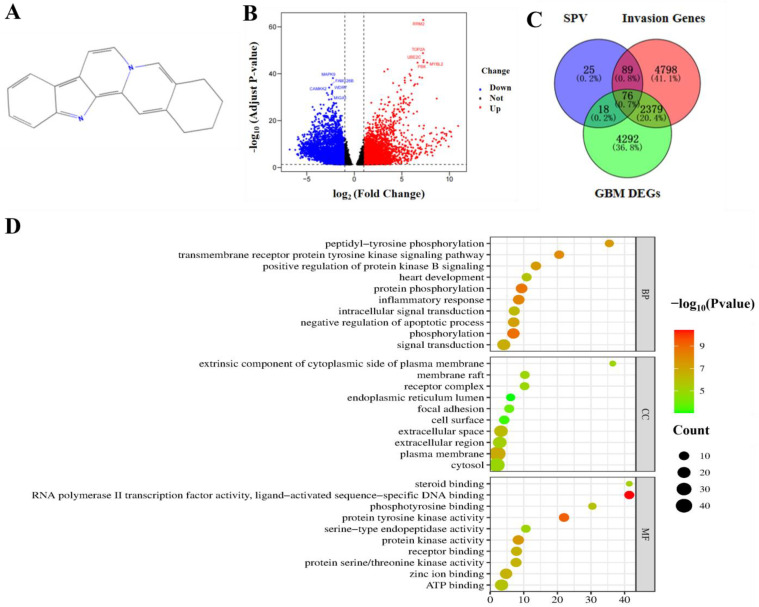

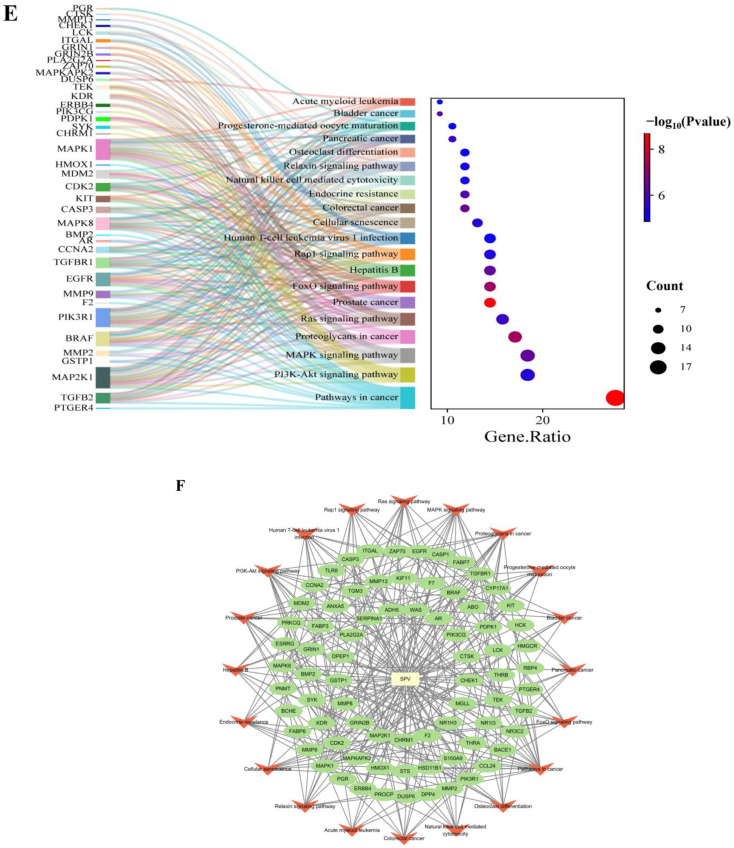
Network pharmacology analysis of SIGI. (**A**) Molecular structure of SPV. (**B**) Volcano plot of GBM DEGs. (**C**) Venn diagram of the overlapping targets of SPV, GBM DEGs, and invasion-related genes. (**D**) The GO enrichment analysis in BP, CC, and MF related to the targets of SIGI. (**E**) The KEGG enrichment analysis of the targets of SIGI. (**F**) Compound-disease-target network of SIGI. Yellow nodes indicate the SPV, red nodes denote the 20 signaling pathways screened from the KEGG analysis, and green nodes symbolize the overlapped target of GBM DEGs, targets of SPV, and invasion-related genes.

The chemical structure of SPV downloaded from PubChem is displayed in Figure 2A [17]. To find the targets of SIGI, 208 targets of SPV were identified from ChEMBL, PharmMapper, and Swiss Target Prediction. All 7342 invasion-related genes were identified from GenCards, OMIM, and Drugbank. A total of 6765 DEGs were obtained, which include 3413 up- and 3352 downregulated genes (Figure 2B). Intersection of the above sets had 76 genes, which were regarded as potential targets of SIGI (Figure 2C). Information on the 76 genes are provided in Supplementary Table S1. The GO enrichment analysis was then performed on 76 targets, revealing potential therapeutic pathways and showing the top 10 terms that were significantly enriched in the biological process (BP), cell component (CC), and molecular function (MF) categories (Figure 2D). The results showed that ‘peptidyl-tyrosine phosphorylation’, ‘extrinsic component of cytoplasmic side of plasma membrane’, and ‘steroid binding’ were the most significantly enriched items of BP, CC, and MF, respectively. Additionally, through KEGG enrichment analysis, the result indicated that the 76 targets were mainly involved in 123 pathways, and the top 10 signaling pathways were visualized according to the *p*-value. As shown in Figure 2E, the ‘PI3K-Akt signaling pathway’ and ‘MAPK pathway’ comprised the larger number of targets. To elucidate multiple pathways and targets involved in SIGI, we utilized the Cytoscape 3.10.1 to visualize a compound-targets-pathway network comprising 76 targets, 20 pathways, and 290 edges (Figure 2F).

### 2.2. Construction and Validation of a Prognostic Model for Patients with GBM in the TCGA and GEO Cohort

**Figure 3:**
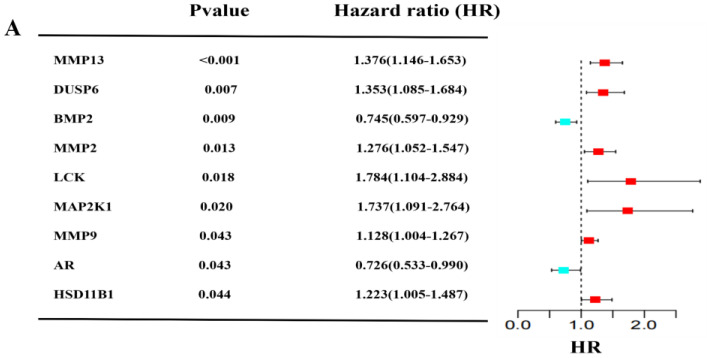

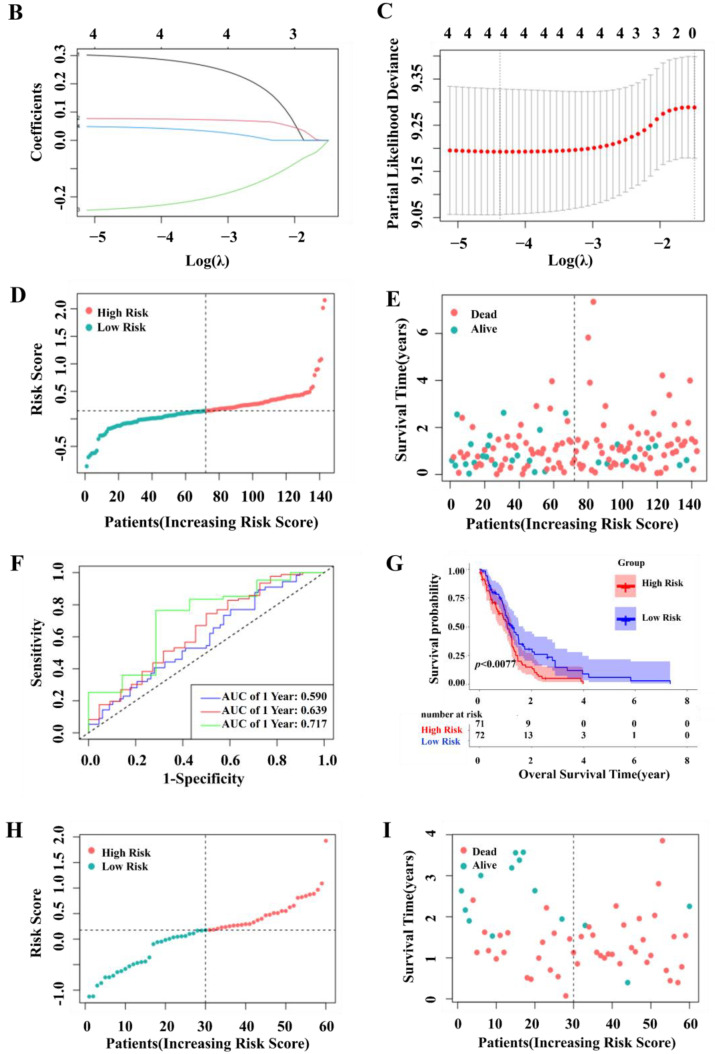

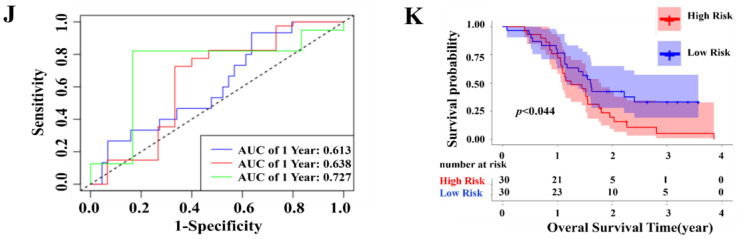
Construction of the prognostic model with TCGA and GEO samples. (**A**) Univariate Cox regression analysis of 76 genes with *p* < 0.05 in TCGA_GBM database; hazard ratio (HR) represents the ratio by which a factor affects survival (HR < 1: protective roles; HR > 1: adverse roles; HR = 1: make no difference). (**B**) LASSO coefficient profiles of the 4 targets of SIGI with non-zero coefficients in the TCGA_GBM database; (**C**) LASSO regression with the screening of optimal parameters (lambda) obtained 4 prognostic genes. The distribution and median value of the SIGI risk score in the TCGA samples (**D**) and the GEO samples (**H**). The distributions of survival status and SIGI risk scores in the TCGA samples (**E**) and the GEO samples (**I**). The AUC of time-dependent ROC curves of the TCGA samples (**F**) and the GEO samples (**J**). The Kaplan–Meier curves of the TCGA samples (**G**) and the GEO samples (**K**).

Figure 3A of the univariate Cox analysis shows that nine targets are significantly related to the SIGI. *BMP2* and *AR* play protective roles in GBM patients (HR < 1) while others have adverse roles (HR > 1). Then, the relationship between the nine targets and overall survival (OS) was explored, high expressions of *DUSP6*, *MMP13*, and *MMP2* were found to be significantly related to poor OS in GBM patients, and *BMP2* had the opposite result (Supplementary Figure S1). Meanwhile, the expressions of *DUSP6*, *BMP2*, *MMP13*, and *MMP2* were higher in GBM patients compared with normal samples (Supplementary Figure S2). Next, the predictive characteristics of the four genes were analyzed by the LASSO (Figure 3B,C). We further combined the survival curves of each gene and selected four genes with statistical differences to construct the prognosis model by LASSO-penalized Cox regression analysis. Lasso regression uses L1 norm to carry out contraction penalty, which can compress some less important variable coefficients to 0 so as to reduce the number of genes obtained via Cox regression. The Lasso regression showed that when Log(λ) was −4.4, the model had the best fitting effect, and these four genes were all important variables included in the model construction (coefficient ≠ 0). Four genes of SIGI were screened out based on the optimal value of λ and used to establish a prognostic model. In Figure 3D,E, we used the median risk scores to divide GBM samples into the high- and low-risk groups, and more patients died in the high-risk group than in the low-risk group. Then, Kaplan–Meier (KM) analysis was used to assess the overall survival of two groups in TCGA samples, which shows that the difference in OS between the high-risk group and the low-risk group was statistically significant (*p* < 0.05) (Figure 3G). The performance of the prognostic models was assessed by receiver operating characteristic (ROC) curves and the area under the curve (AUC) of 1-, 2-, and 3-years was 0.590, 0.639, and 0.637, respectively (Figure 3F). According to the risk scoring formula of TCGA samples, the patients in GEO were divided into high and low-risk group to external verification (Figure 3H,I). The Kaplan–Meier (KM) analysis also revealed the same result as the TCGA samples (*p* < 0.05) (Figure 3K). Additionally, the AUC of 1-, 2-, and 3-year was 0.613, 0.638, and 0.727, respectively (Figure 3J). These results suggested that the prognostic model based on *DUSP6*, *MMP2*, *MMP13* and *BMP2* genes has good stability.

### 2.3. Independent Prognostic Value of the Four-Gene Prognostic Model and Molecular Docking

**Figure 4:**
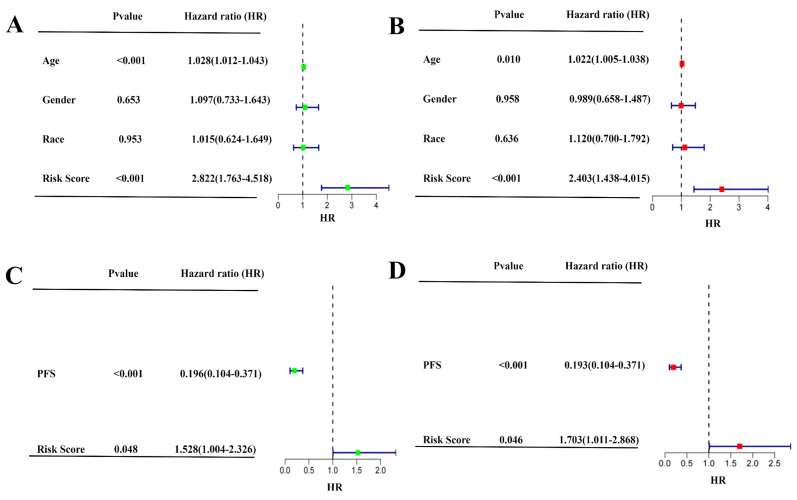
Univariate and multivariate Cox regression analyses concerning OS in the TCGA samples (**A**,**B**) and the GEO samples (**C**,**D**). Hazard ratio (HR) represents the ratio by which a factor affects survival (HR < 1: protective roles; HR > 1: adverse roles; HR = 1: make no difference).

**Figure 5:**
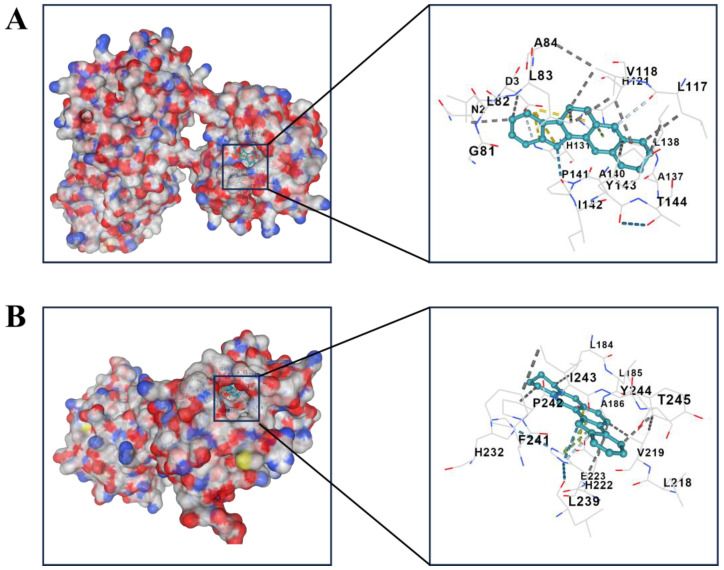

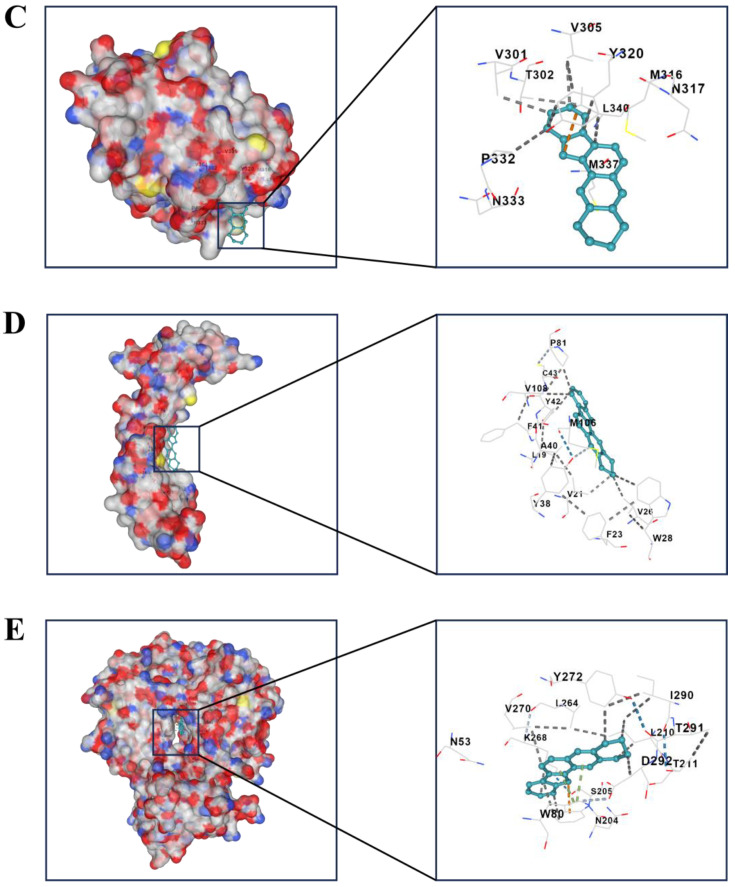
The results of molecular docking (**A**–**E**). The docking modes of SPV with MMP2, MMP13, DUSP6, BMP2, AKT1.

To further confirm whether the SIGI risk score of prognostic mode based on four genes could be applied as an independent prognostic factor for SIGI, the predictive value of risk score was evaluated through univariate and multivariate Cox analyses. The univariate Cox analysis showed that GBM patients in TCGA and GEO were significantly related to the risk score. In TCGA and GEO samples, the hazard ratios (HR) were 2.822 and 1.528, respectively, which had statistical significance in both (Figure 4A,C). The results of the multivariate Cox analysis demonstrated that the risk score remained an independent predictor for OS, which was adjusted for other confounding variables. The hazard ratios (HR) were 2.403 and 1.703 in TCGA samples and GEO samples, respectively, which had statistical significance in both (Figure 4B,D). Then, molecular docking was performed between the four genes and SPV (Figure 5A–E). The complementarity between the four genes and SPV was evaluated by the Vina Score (negative correlation between the Vina Score and stability). Among them, the Vina Scores of DUSP6, BMP2, MMP2, MMP13, and AKT1 with SPV were −7.8, −6.5, −10.4, −10.4, and −10.9 (Table 1). Molecular docking of AKT1, MMP2, and MMP13 with corresponding inhibitors was performed. The results showed that the Vina Scores of MMP2 and MMP13 with their respective inhibitors were higher than that of SPV, while the Vina Score of AKT1 with its inhibitors was lower than that of SPV (Supplementary Figure S3).

### 2.4. SPV Efficiently Inhibits Invasion, Migration, and Adhesion on GBM Cells

**Figure 6:**
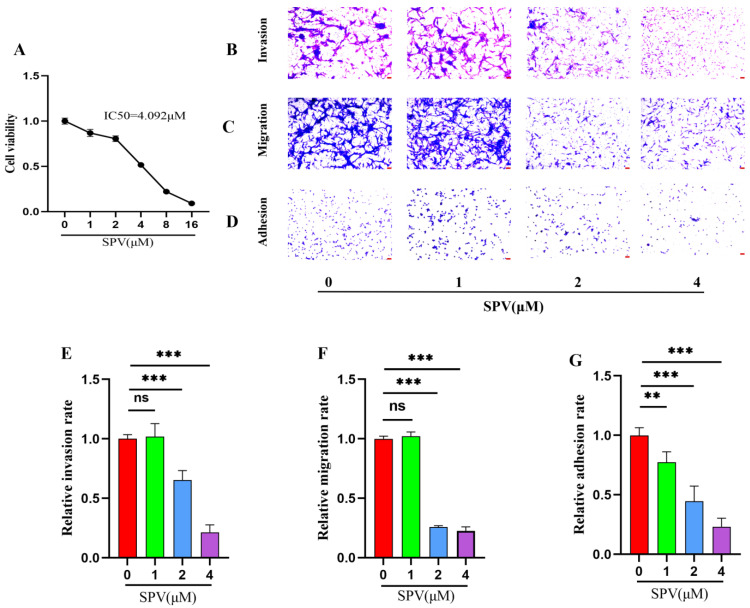
Sempervirine efficiently inhibits invasion, migration, and adhesion in U87 cells. (**A**) After SPV intervention for 48 h, cell viability was measured by CCK8 assay. (**B**,**E**) Transwell invasion assay was treated with SPV for 36 h in U87 cells. (**C**,**F**) Transwell migration assay of U87 cells was treated with SPV for 36 h. (**D**,**G**) Effect of SPV on the adhesion to Matrigel-coated plate after 1 h exposure. The cells were stained with crystal violet to be photographed (×200) and calculated using Image J (1.52i) software (n = 5). Data are presented as the Mean ± SD (n = 5). ^ns^ *p* > 0.05, ** *p* < 0.01 and *** *p* < 0.001 compared with the control group.

**Figure 7:**
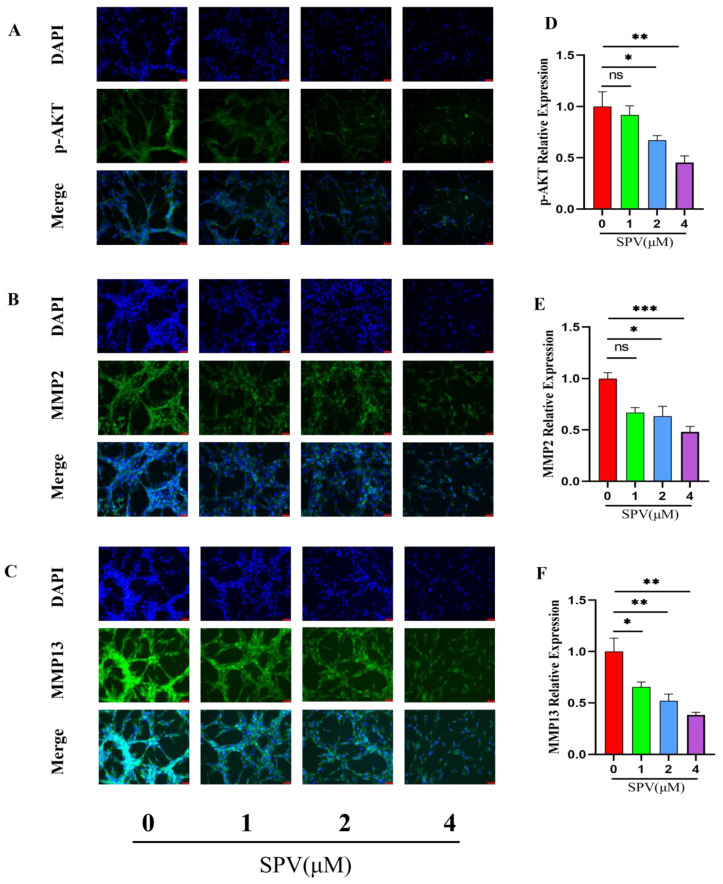

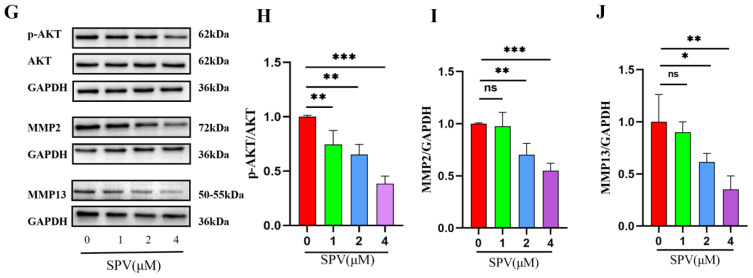
SPV regulates p-AKT, MMP2, and MMP13 expression in U87 cells. (**A**–**F**) P-AKT, MMP2, and MMP13 was measured by immunofluorescence analysis of U87 cells treated with the series concentrations of SPV for 48 h. The cells were photographed (×200) and calculated by Image J (1.52i) software (n = 5). (**G**–**J**) P-AKT, MMP2, and MMP13 was determined by Western blotting. Data are presented as the Mean ± SD (n = 3). ^ns^ *p* > 0.05, * *p* < 0.05, ** *p* < 0.01, and *** *p* < 0.001 compared with the control group.

As Figure 6A of CCK-8 results, the cell viability of the U87 cells was significantly reduced through the series concentrations (0, 1, 2, 4, 8, and 16 μM) of SPV intervention, and SPV inhibited proliferation of U87 cells in a dose-dependent manner at 48 h. The IC50 was 3.942 ± 0.232 μM, and then doses less than IC50 were selected to intervene in the U87 cells. The impact of SPV on migration, invasion, and adhesion in the U87 cells were detected via a transwell assay and cell adhesion assay. The results showed that after an SPV intervention of 48 h, the rates of invasion, migration, and adhesion were gradually reduced (Figure 6B–G). The expressions of the three targets were analyzed by immunofluorescence staining to determine whether SPV could inhibit the expressions of p-AKT, MMP2, and MMP13 in the U87 cells. As shown in Figure 7A–F, immunofluorescence staining of p-AKT, MMP2, and MMP13 was markedly decreased following SPV treatment. Further, Western blot was used to detect the expressions of p-AKT, MMP2, and MMP13 proteins. As shown, SPV could inhibit the expressions of p-AKT/AKT, MMP2, and MMP13 in a dose-dependent manner (Figure 7G–J). The same results can be observed in U251 cells (Supplementary Figures S4 and S5).

### 2.5. Regulation of AKT Signaling Affects Invasion, Migration and Adhesion in GBM Cells

**Figure 8:**
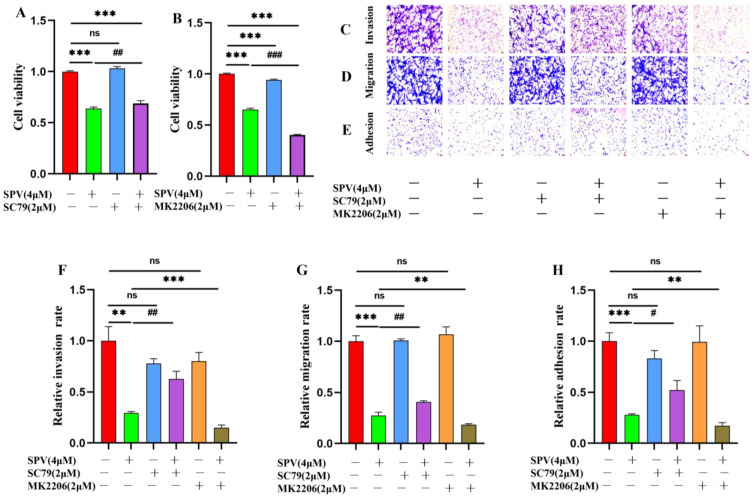
SPV efficiently inhibits invasion, migration and adhesion in U87 cell through regulating *AKT* phosphorylation. (**A**,**B**) After SC79 and MK2206 intervention for 2 h, U87 cells treated with SPV for 48 h. Cell viability was determined by CCK8. (**C**,**F**) After SC79 and MK2206 intervention for 2 h, Transwell invasion assay of U87 cells treated for 36 h. (**D**,**G**) After SC79 and MK2206 intervention for 2 h, Transwell migration assay of U87 cells treated for 36 h. (**E**,**H**) Effect of SPV on the adhesion to Matrigel coated plate after 1 h exposure. The cells were photographed (×200) and calculated by Image J (1.52i) software (n = 5). Data are presented as the Mean ± SD (n = 5). ^ns^ *p* > 0.05, ** *p* < 0.01 and *** *p* < 0.001 compared with the control group. # *p* < 0.05, ## *p* < 0.01 and ### *p* < 0.001 compared with SPV group.

To verify whether SPV mediates the inhibitory effect on GBM invasion, migration, and adhesion through *AKT* targets, U87 cells were pre-cultured with MK2206 (2 μM) or SC79 (2 μM) before a culture with 4 μM SPV for 48 h. Cell proliferation was observed by CCK8 assay, and the results showed that compared with the group cultured with SPV, cell viability was higher in the group that combined SPV with SC79, while cell viability was lower in the group that combined SPV with MK2206, and the cell viability of groups cultured SC79 and MK2206 alone separately showed no difference compared with the control group (Figure 8A,B). Next, a transwell assay was performed to simulate the phenomenon of cells across ECM to distant locations to observe the changes in invasion and migration ability of U87 cells. Both transwell invasion and transwell migration assays showed that compared with the group cultured with SPV, activation of AKT increased the invasion and migration ability of U87 cells, while inhibition of AKT inhibited the invasion and migration ability of U87 cells, and the invasion and migration ability of U87 cells cultured SC79 and MK2206 alone separately did not differ from the control group (Figure 8C,D,F,G). Then, Cell adhesion assay was used to simulate the ability of tumor cells to migrate to distant locations and form metastases to observe the change in adhesion ability of U87 cells. The results also showed that compared with the group cultured with SPV, activation of AKT increased the adhesion ability of U87 cells, while inhibition of AKT had the opposite effect (Figure 8E,H).

**Figure 9:**
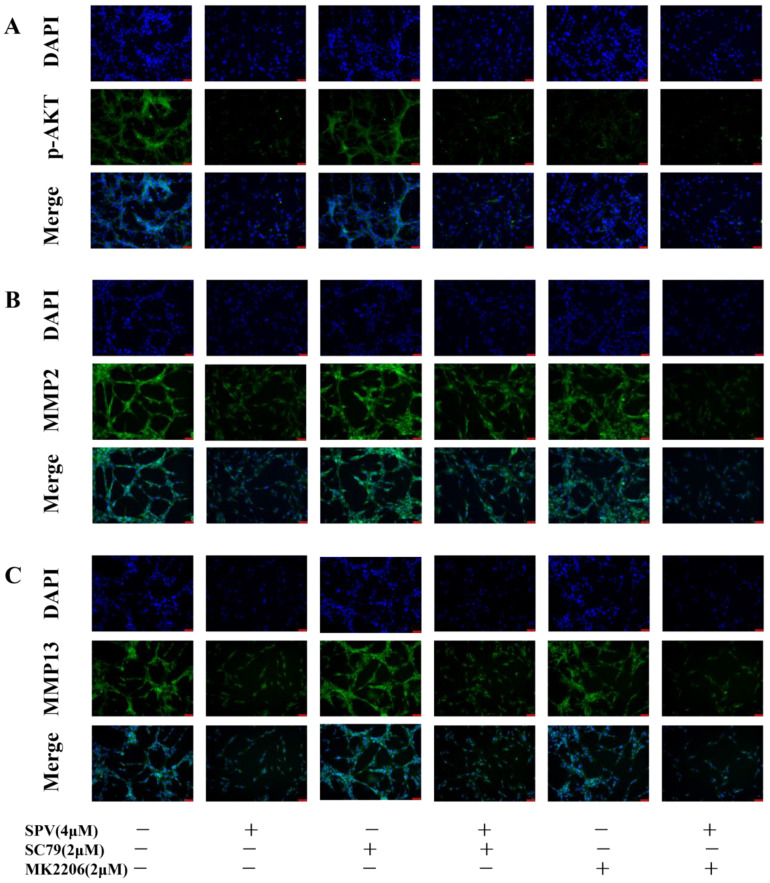

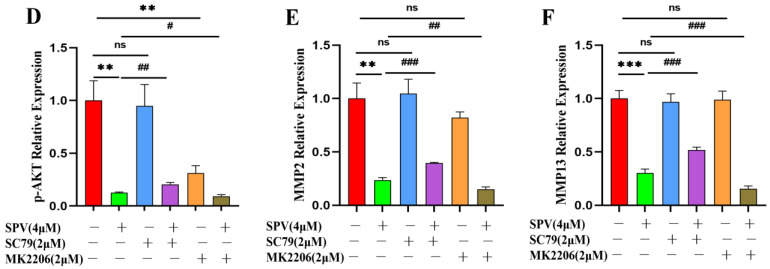
SPV affects the expression levels of MMP2 and MMP13 via regulating AKT phosphorylation in U87. (**A**–**F**) After SPV intervention for 48 h, immunofluorescence analysis was used to measure p-AKT, MMP2, and MMP13 of U87cells. The cells were photographed (×200) and calculated using Image J (1.52i) software (n = 5). Data are presented as the Mean ± SD (n = 5). ^ns^ *p* > 0.05, ** *p* < 0.01, and *** *p* < 0.001 compared with the control group. # *p* < 0.05, ## *p* < 0.01 and ### *p* < 0.001 compared with SPV group.

**Figure 10:**
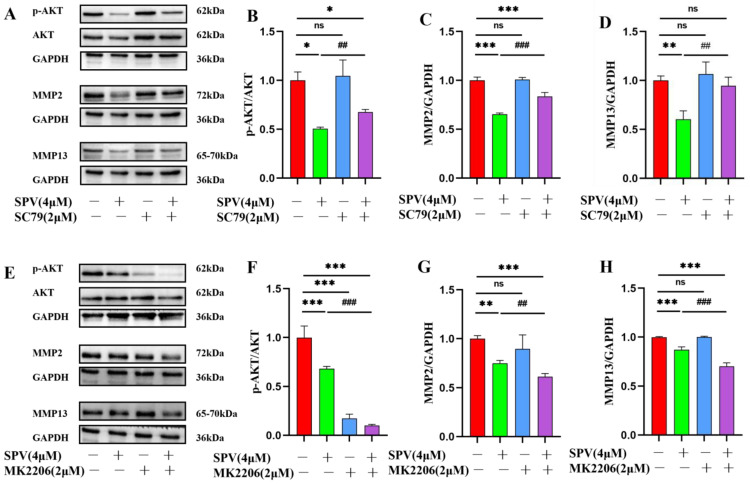
SPV affects the expression levels of MMP2 and MMP13 in U87 via regulating AKT phosphorylation. (**A**–**D**) SC79 was added to cells 2 h before SPV intervention. After 48 h, Western blot was used to analyze p-AKT, MMP2, and MMP13 in U87. (**E**–**H**) MK2206 was added to cells 2 h before SPV intervention. After 48 h, p-AKT, MMP2, and MMP13 were analyzed via Western blotting. Data are presented as the Mean ± SD (n = 3). ^ns^ *p* > 0.05, * *p* < 0.05, ** *p* < 0.01, and *** *p* < 0.001 compared with the control group. ## *p* < 0.01, and ### *p* < 0.001 compared with SPV group.

To further explore the involvement of the *AKT* target in mediating the influence of SPV on MMP2 and MMP13, the expressions on cells were examined by immunofluorescence staining and Western blot. The results of immunofluorescence staining showed that when SC79 and MK2206 were used alone, the expressions of *MMP2* and *MMP13* did not change significantly with the control group. Compared with the group cultured with SPV, SC79 could increase the fluorescence intensity of MMP2 and MMP13 in the presence of SPV, while MK2206 had the opposite effect (Figure 9A–F). The results of Western blot were the same as immunofluorescence staining. When SC79 and MK2206 were used alone, there was no significant change in the expressions of *MMP2* and *MMP13* compared with the control group. In the case of SPV intervention, SC79 could increase the expressions of MMP2 and MMP13 (Figure 10A–D), and when AKT was inhibited, the effect of SPV was enhanced (Figure 10E–H). This finding confirmed that SPV inhibits the expressions of MMP2 and MMP13 proteins via inhibiting AKT phosphorylation, thereby affecting the invasion ability of GBM cells.

## 3. Discussion

GBM, the WHO IV glioma, is the most malignant and invasive glioma, which accounts for 58% of glioma patients and 48% of malignant tumors in the central nervous system [18,19]. One of the clinical hallmarks of GBM is its diffuse growth, which often invades cerebral lobes and deep brain structures, even extending to the contralateral brain [19,20]. Although GBM patients in recent years have been able to receive a full range of systemic treatments, such as radiation, chemotherapy, and supportive care, the growth rate of tumor cells is still high, which results in a disease course of 3 to 6 months [21]. At the same time, due to GBM invasion, patients are often accompanied by a series of complications that lead to an increased burden of life. Radiation therapy, to prevent damage to healthy tissues, will miss tumor cells that invade other parts of the brain tissue because of cell invasion. Similarly, chemotherapy is administered systemically and enters the tumor through the blood vessels, so it will also be limited by tumor spread [22,23]. Due to the aggressive nature of GBM, it is desirable to combine drugs to inhibit the invasion in clinical practice. However, there have been many small molecule inhibitors of cancer cell invasion that have shown success preclinically, though fewer clinically [24], which has led to the development of drugs targeting the invasiveness of GBM urgently.

SPV is a potential alkaloid component with multiple tumor-inhibitory effects, and it has been confirmed that SPV can significantly inhibit cell proliferation. However, the pharmacodynamics of SPV on GBM invasion has not been reported. This study found that when the dose of SPV below IC50 interferes with GBM cells, the rate of invasion, migration, and adhesion in GBM cells are significantly reduced, which may have good prospects for the inhibition of GBM invasion. However, the mechanism of the inhibition of SPV on GBM invasion is still not clear. Therefore, exploring the mechanism of SIGI is the focus of this study.

The related pathways and 76 potential targets of SPV inhibiting GBM invasion were initially screened using network pharmacology, and the results of GO analysis and KEGG analysis were also consistent with our previous studies that SPV inhibited the *AKT* signaling pathway to induced autophagy and apoptosis of glioma cells. GBM is a highly aggressive tumor, and the cause of death in most patients is recurrent death due to incomplete tumor resection [25]. Therefore, we determined the relationship between 76 genes and the survival time of patients via univariate Cox proportional hazards regression analysis and further identified the target of SPV to inhibit GBM invasion. The results showed that nine genes were associated with the survival time of patients, among which *BMP2* and AR were protective factors in glioma patients, and the other seven genes were risk factors. All of these nine genes have been reported to affect the invasion and metastasis ability of tumor cells. Still only *BMP2*, *MMP13*, *MMP2*, *LCK, MMP9*, and *AR* have been reported to affect the glioma invasion. For example, the administration of recombinant human bone morphogenic protein-2(rhBMP-2) reduces the aggressiveness of C6 glioma models in vivo [26]. Silencing *circ-ASPH* can reduce the AR expression level to weaken proliferation, clonal formation, and the invasion of glioma cells [27]. The effects of *BMP2* and *AR* on glioma reported in the literature are consistent with the results of this study showing that *BMP2* and AR are protective factors in glioma patients. Matrix metalloproteinases (MMPs) are calcium- and zinc-dependent endopeptidases, which can degrade and remodel extracellular Matrix (ECM) proteins to induce tumor invasion and migration [28]. Overexpression of *MAGI1* can affect AKT, MMP2, and MMP9 and inhibit the invasion and migration of glioma cells [29]. *RSU-1* is significantly upregulated in more aggressive glioma cell types, and silencing *RSU-1* can reduce STAT6 and MMP13 to inhibit invasion [30]. It has also been reported that small molecule inhibitors (LCK-I) are used to inhibit LCK phosphorylation, thereby affecting downstream target phosphorylation to inhibit cell migration [31].

Protein kinase B (*AKT*) includes three types: *AKT*1, *AKT*2, and *AKT*3. *AKT* regulates various biological processes such as ECM remodeling, epithelial–mesenchymal transition (EMT), and cell cycle through phosphorylation of downstream effector molecules, thus promoting tumor formation, invasion, and migration [32]. For example, the highly expressed *TPX2* in HCC might promote tumor cell invasion via activating AKT signaling and subsequently increasing MMP2 and MMP9 expression [33]. Ginsenoside Rh2 may inhibit GBM invasion and migration through inhibiting *AKT*-mediated *MMP13* activation [34]. In glioma, inhibition of AKT1 phosphorylation at Ser473 has been reported to inhibit GBM invasion. For example, Tetrandrine inhibited metastasis-related proteins, such as p-AKT (Ser473), MMP2, and MMP9, which can inhibit GBM invasion [35]. The *CK1* gene promoted cell proliferation and invasion through the phosphatidylinositol 3 kinase/matrix metalloproteinase 2(AKT-MMP2) signaling pathway [36]. Then, we further verified the binding effect of SPV with DUSP6, BMP2, MMP2, MMP13, and AKT1 via molecular docking. It is worth noting that although the four-genes model was also verified in various ways to show that the model fits well. The results of molecular docking showed that SPV only had a high binding effect with MMPs, indicating that the combination of network pharmacology and bioinformatics has advantages for core target screening.

To further confirm the results obtained through network pharmacology and bioinformatics, we observed the mechanism of SPV to inhibit GBM invasion in vitro. A dose less than IC50 was selected to intervene in the cell, which avoids the induction of apoptosis and autophagy. A transwell assay and cell adhesion assay were used to observe the effect of SPV on GBM invasion, migration, and adhesion. The transwell assay was coated with Matrigel, which is a common test for tumor invasiveness [37]. For the detection of tumor migration ability, wound healing assay is a common method used [38]. However, with the wound healing assay of U87 and U251 cells used in this study, it was found that U87 cells were prone to form aggregation, and it was easy to cause a large area of cells outside the blank area to shed when artificial blank areas were made, which affected the results of the test. Therefore, a transwell assay without Matrigel was used to detect tumor migration ability. The possible relationship between morphology and cell aggressiveness was evaluated via microscopy, and it was found that U87 belonged to the more aggressive GBM cells, and the U87 cells were selected to verify whether SPV inhibited GBM invasion via regulating *AKT* phosphorylation to affect the expressions of *MMP2* and *MMP13*. Meanwhile, the anti-GBM invasion activity of SPV observed in in vitro studies was also significantly higher than most natural compounds, such as dihydroartemisinin (IC50 > 100 μM) [39], fraxetin (IC50 > 100 μM) [40], and Oleanolic acid (IC50 > 10 μM) [41], which indicate that SPV has the prospect of developing patent drugs in glioma invasion treatment.

In our previous study, it was confirmed that SPV can significantly induce autophagy and apoptosis in glioma cells to anti-tumor, but it has only been preliminarily confirmed that SPV can induce the phosphorylation of *AKT* target to anti-tumor, and how SPV further affects cell apoptosis, autophagy, and invasion through *AKT* is still unknown. In the previous study, we also determined the equilibrium solubility and oil–water partition coefficient of SPV to guide the development of its corresponding dosage forms, and successfully prepared SPV in chitosan thermosensitive microemulsion-based hydrogel and microemulsion. No significant ciliary toxicity and nasal mucosal irritation were observed through the corresponding safety evaluation, which is expected to improve the efficacy of nasal administration in glioma patients [42,43,44]. In this study, we further found and verified that SPV can inhibit GBM invasion via regulating *AKT* phosphorylation to affect the expressions of *MMP2* and *MMP13*, which lays a foundation for future drug development of SPV.

## 4. Materials and Methods

### 4.1. Chemicals and Reagents

Sempervirine (SPV, Percent Purity, ≥98.0%, 63726-006A2) was provided by 3408 Laboratory, College of Pharmacy, Fujian University of Traditional Chinese Medicine; CCK-8 (K1018), Phosphatase Inhibitor Cocktail (K1015) was purchased from ApexBio Technology (Shanghai, China); MK2206 (MK, SF2712), SC79 (SC, SF2730), Triton X-100 (ST1723-100 mL), and Cell Complete Lysis Buffer for Western and IP (P0037-100 mL) were purchased from Beyotime (Shanghai, China); GAPDH Polyclonal antibody (10494-1-AP), Phospho-*AKT*(Ser473) Monoclonal antibody (66444-1-lg), *AKT* Polyclonal antibody (10176-2-AP), *MMP13* Polyclonal antibody (18165-1-AP), and *MMP2* Polyclonal antibody (10373-2-AP) were purchased from Proteintech (Wuhan, China). Western Blot Fast Stripping Buffer (PS107) and Protein Free Rapid Blocking Buffer (1×) (PS108P) were purchased from epizyme (Shanghai, China); Bovine Serum Albumin (GC305010-25g), electrophoresis buffer (G2081-1L) and transfer buffer (G2028-1L) were purchased from Servicebio (Wuhan, China); Goat Anti-Rabbit IgG H&L (Alexa Fluor^®^ 488) (ab150077) and Goat Anti-Mouse IgG H&L (Alexa Fluor^®^ 488) (ab150113) were purchased from Abcam (Shanghai, China).

### 4.2. Cell Culture

U87 cells and U251 cells, the human GBM cell lines, were obtained from Wuhan Pricella Biotechnology Co., Ltd. (Wuhan, China) and cultured, respectively, in Minimum Essential Medium contained 10% FBS (Sigma, St. Louis, MO, USA, F8318-500ML) and Dulbecco’s Modified Eagle Medium contained 10% FBS (Sigma, St. Louis, MO, USA, F8318-500ML) in 5% CO_2_ at 37 °C.

### 4.3. Datasets and Genes Participating in GBM

The clinical and RNA transcription data of GBM patients from TCGA database were downloaded, which included 5 normal samples and 144 GBM samples [45]. The clinical and RNA transcription data of GBM patients from GEO database (Series: GSE74187, Platform: GPL6480) [46] included 60 GBM samples, which were used to conduct external verification for a prognostic model of four genes.

### 4.4. Collection of Invasion and SPV-Related Target Genes

Targets of SPV were collected from public databases, including ChEMBL [47], PharmMapper [48], and SwissTarget Prediction [49]. Invasion-related genes were collected from public databases, including GeneCards, Online Mendelian Inheritance in Man (OMIM) [50], and DrugBank Online [51]. After being standardized into gene symbol by the UniProt database (https://www.uniprot.org/, accessed on 29 November 2023) [52], DEGs, invasion-related genes, and targets of SPV were intersected and visualized using Venny 2.1.0.

### 4.5. GO, KEGG Pathway Enrichment Analysis, and Compound-Targets-Pathway Network Construction

The GO and KEGG enrichment of the overlapping genes was performed using the DAVID database (https://david.ncifcrf.gov/summary.jsp, accessed on 28 November 2023) [53]. Then, the complex interactions of the compound-targets-pathways network were visualized by Cytoscape (3.10.1) [54].

### 4.6. Constructing and Validating a Prognostic Gene Signature for the Inhibition of SPV on GBM Invasion

According to the limit of *p* < 0.05 and |log2 Fold Change (FC)| > 1, we obtained the DEGs between tumor and normal patients in TCGA samples using the “limma” R package. Then, the targets of SIGI were distinguished by taking an intersection of the DEGs, invasion-related genes, and SPV targets. To screen the targets of SIGI with prognostic significance, the targets were used for analysis via univariate Cox proportional hazards (COX) regression analysis using the “survival” R package. Targets of SIGI with prognostic significance were further analyzed for their relationship with overall survival (OS), and candidate targets of SIGI were identified (*p* < 0.05) via gene expression profiling interactive analysis (GEPIA) (http://gepia.cancer-pku.cn, accessed on 28 November 2023) [55]. To identify a novel prognostic signature on the basis of candidate targets of SIGI, LASSO-penalized Cox regression analysis using the “glmnet” R package was applied to construct a prognostic model. According to the penalty parameter (λ) by ten-fold cross-validation following the minimum criteria, a prognostic model with four genes was established successfully. The risk score was calculated using the following formula. Based on the median risk score, TCGA samples were classified into high- and low-risk groups, which were regarded as training cohorts. GEO samples on the basis of the corresponding coefficient of genes from TCGA samples were classified into high- and low-risk groups, which were regarded as validation cohorts.

(1) $$\mathrm{SIGI}\;\mathrm{Risk}\;\mathrm{Score}=\sum_{i=1}^{n}Coef_{i}\times x_{i}$$

### 4.7. Evaluation of the Gene Signature Accuracy

We assessed the predictive efficacy of the four-genes model using the receiver operating characteristic (ROC) curve, and the difference in overall survival (OS) between two groups was further tested using a Kaplan–Meier analysis. To verify whether risk score was an independent prognostic factor in patients with GBM, univariate and multivariate Cox regression analyses were performed.

### 4.8. Molecular Docking

The molecular structures of the four-genes model, which included *DUSP6* (1MKP), *BMP2* (1REU), *MMP2* (7XGJ), *MMP13* (4JPA), and *AKT1* (7NH5), were downloaded from the Protein Data Bank (PDB) [56]. Finally, the CB-Dock2 (https://cadd.labshare.cn/cb-dock2/php/index.php, accessed on 4 January 2024) was run to simulate the molecular docking between SPV and the candidate targets [57].

### 4.9. Cell Viability Assay

The cells were pre-inoculated in 96-well plates for 24 h and then treated with SPV of 0, 2, 4, 8, and 16 μM. After 48 h, cell viability was measured via CCK-8, and the optical density (OD) ratio of treated to non-treated control cells was regarded as cell viability in relation to the non-treated control. Sempervirine was dissolved in DMSO and then diluted with cell culture medium. In all experiments, the final concentration of DMSO was much less than 0.1% (*v*/*v*). Cells treated with equal amounts of DMSO were used as controls.

### 4.10. Transwell Assay

Matrigel (dilution ratio, 1:8, Corning, NY, USA) was coated on the interior surface of the upper chamber and incubated for 30 min for the cell invasion assay. The cells (2.5 × 10^5^ Cells/mL) were re-suspended in serum-free neurobasal medium with a corresponding concentration of SPV and then inoculated in the upper chamber at a volume of 200 μL. Finally, 600 μL neurobasal medium, which contained 20% FBS on basis of the neurobasal medium in the upper chamber, was in the lower chamber. After 36 h, the membrane of the upper chamber was fixed with 4% paraformaldehyde for 15 min. After removing noninvaded cells, 0.1% crystal violet staining solution was used to stain cells for 1 h and washed with distilled water. In contrast to the procedure of cell invasion, only the cell migration assay did not use Matrigel to coat the membrane surface of the upper chamber. The situation of cell invasion and migration was observed via microscope, and the Image J (1.52i) software was used for the quantitative analysis to calculate the ratio of cell invasion and migration.

### 4.11. Adhesion Assay

The cells treated with SPV for 48 h were inoculated in 24-well plates coated with Matrigel (dilution ratio, 1:8, Corning, USA) to incubate for 1 h. After the nonadhesive cells were cleaned by PBS, the remaining cells were fixed with 4% paraformaldehyde for 15 min and then stained with 0.1% crystal violet staining solution. The situation of cell adhesion was observed via microscope, and the Image J (1.52i) software was used for quantitative analysis to calculate the ratio of cell adhesion.

### 4.12. Immunofluorescence Staining

The cells treated with SPV for 48 h were fixed with cold 4% PFA for 10 min and permeated in 0.5% Triton X-100. To block cells for 1 h, 5% BSA was used, and then primary antibodies including p-*AKT* (1:100), *MMP2* (1:100), and *MMP13* (1:100) were added, respectively, to incubated with cells overnight at 4 °C. The fluorescent secondary antibody was added to incubated cells for 1 h at room temperature. Finally, we stained the nucleus with DAPI and took fluorescence images under a fluorescence microscope. The fluorescence intensity of cells was quantitatively analyzed using Image J.

### 4.13. Western Blot Assay

The cells treated with SPV for 48 h were lysed by adding Cell Complete Lysis Buffer for Western and IP to extract total cellular protein. Then, we used the BCA method to quantify the protein. Approximately 10 μg of protein was separated by SDS-PAGE and transferred to PVDF membranes. The membranes were sealed with protein-free rapid blocking buffer, which was incubated overnight at 4 °C with primary antibody (P-*AKT* (1:2000), GAPDH (1:50,000), *AKT* (1:2000), *MMP2* (1:1000), and *MMP13* (1:1000)), respectively. After incubation of the HRP-coupled second antibody for 2 h, the immunoreactive staining was performed using a chemiluminescence kit and after that visualized utilizing the Bio-Rad ChemiDoc XRS + System.

### 4.14. Statistical Analysis

Results were expressed as the mean ± SD. All in vitro experiments as displayed in the figure legends were analyzed appropriately by Student’s *t* test or one-way ANOVA utilizing IBM SPSS statistics software. Statistical significance was expressed as * *p* < 0.05.

## 5. Conclusions

In this study, we first predicted the potential targets of SPV inhibition of GBM invasion through bioinformatics analysis. Cell biology analysis shows that SPV significantly inhibits the proliferation, invasion, metastasis, and potential regulatory effects of *AKT* targets in GBM. By combining an *AKT* activator and inhibitor, it was clarified that SPV inhibits GBM invasion by regulating *AKT* phosphorylation to affect *MMP2* and *MMP13* targets. These findings provide a foundation for future drug development for SPV.

## Figures and Tables

**Table 1:** Vina Score of molecular docking.

Compound	Targets	PDB-ID	Vina Score
Sempervirine	BMP2	1REU	−6.5
	DUSP6	1MKP	−7.8
	MMP2	7XGJ	−10.4
	MMP13	4JPA	−10.4
	AKT1	7NH5	−10.9

## Data Availability

The clinical and RNA transcription data of GBM patients in TCGA and GEO used in this study came from publicly available datasets and were downloaded from the official TCGA websites (https://xenabrowser.net/datapages/, accessed on 28 November 2023), GEO websites (https://www.ncbi.nlm.nih.gov/geo/, accessed on 28 November 2023), and GEPIA (http://gepia.cancer-pku.cn/, accessed on 28 November 2023).

## Supplementary Materials

The following supporting information can be downloaded at: https://www.mdpi.com/article/10.3390/ph17101318/s1, Figure S1. Survival analysis of nine genes that Univariate Cox regression analysis of 76 genes with *p* < 0.05. (A–I). Survival curve of MMP13, DUSP6, BMP2, MMP2, LCK, MAP2K1, MMP9, AR, and HSD11B1; Figure S2. Differential expression analysis of nine genes between normal and tumor patients. (A–I) Box plot of MMP13, DUSP6, BMP2, MMP2, LCK, MAP2K1, MMP9, AR,and HSD11B1 expression between normal and tumor patients; Figure S3. The results of molecular dockingabout AKT1, MMP2, and MMP13 with their inhibitor. (A) The docking mode of MK2206 with AKT1. (B) The docking mode of MMP2-IN-1 with MMP2. (C) The docking mode of MMP13-IN-12 with MMP13; Figure S4. Sempervirine efficiently inhibits the invasion, migration, and adhesion in U251 cells. (A) After SPV intervention for 48h. Cell viability was measured by CCK8 assay. (B,E) Transwell invasion assay were treated with SPV for 36 h in U251 cells. (C,F) Transwell migration assay of U251 cells were treated with SPV for 36 h. (D,G) Effect of SPV on the adhesion to Matrigel coated plate after 1h exposure. The cells were stained with crystal violet to photographed (×200) and calculated by Image J (1.52i) software (n = 5). Data are presented as the Mean ± SD (n = 5). ^ns^ *p* > 0.05, ** *p* < 0.01 and *** *p* < 0.001 compared with the control group; Figure S5. SPV reguletes p-AKT, MMP2 and MMP13 expression in U251 cells. (A–F) P-AKT, MMP2 and MMP13 was measured by immunofluorescence analysis of U251 cells treated with the series concentrations of SPV for 48 h. The cells were photographed (×200) and calculated by Image J (1.52i) software (n = 3). (G–J) P-AKT, MMP2 and MMP13 was determined by Western blot. Data are presented as the Mean ± SD (n = 3). ^ns^ *p* > 0.05, * *p* < 0.05, ** *p* < 0.01 and *** *p* < 0.001 compared with the control group; Table S2. Vina Score of molecular docking about AKT1, MMP2, and MMP3 with their inhibitor; Table S1. Information of 76 genes in TCGA samples.
