# Supplementary material for: Exploring the Mechanism of Sempervirine Inhibiting Glioblastoma Invasion Based on Network Pharmacology and Bioinformatics
Source: Pharmaceuticals (Basel). 2024 Oct 2;17(10):1318. doi: 10.3390/ph17101318 (PMC11510114; doi:10.3390/ph17101318)
Supplement: Supplementary file 1 [file pharmaceuticals-17-01318-s001.zip › Supplemantary Figure S1.pdf]

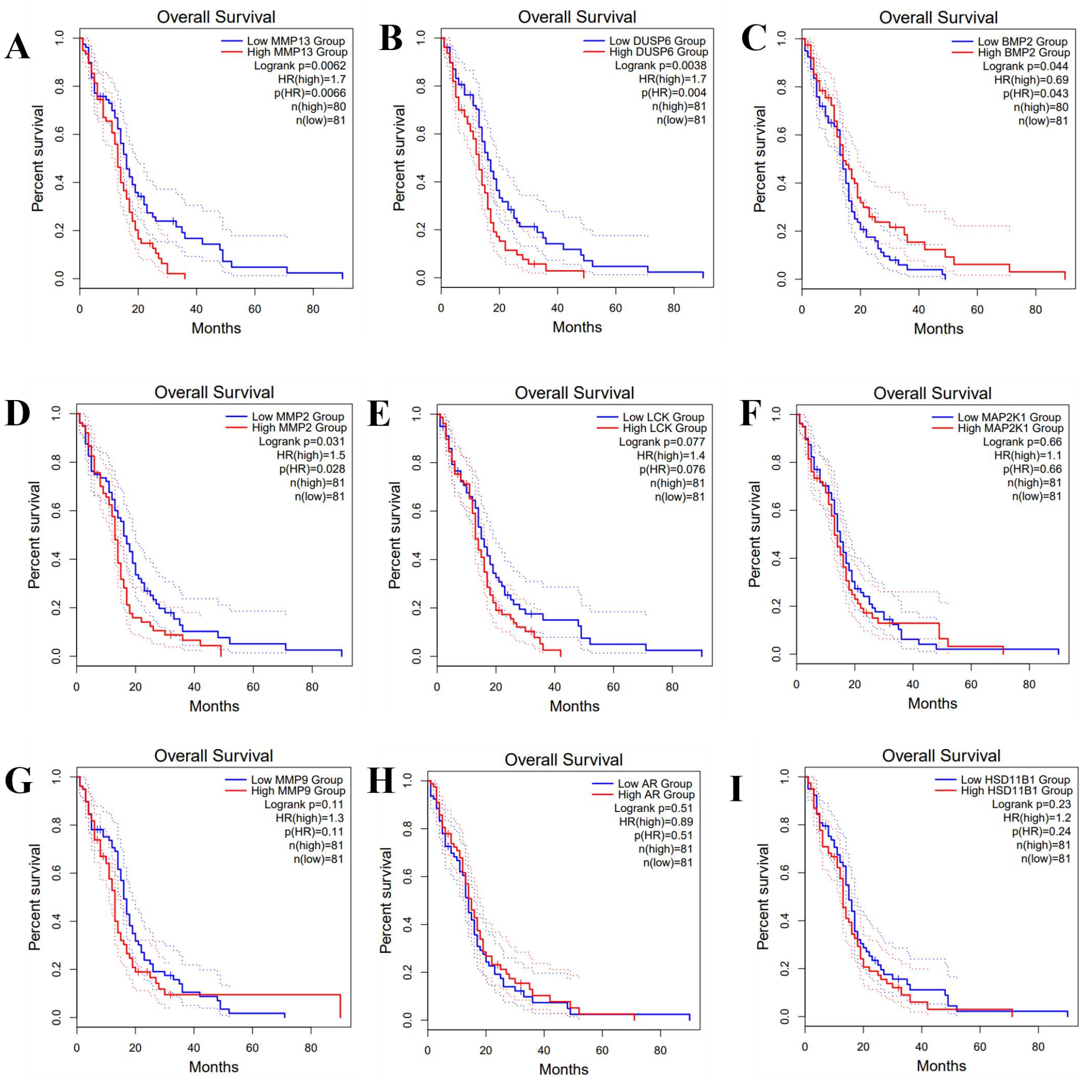

Figure S1. Survival analysis of nine genes that Univariate Cox regression analysis of 76 genes with $P < 0.05$. (A-I). Survival curve of *MMP13, DUSP6, BMP2, MMP2, LCK, MAP2K1, MMP9 ,AR,* and *HSD11B1*
