# Supplementary material for: Exploring the Mechanism of Sempervirine Inhibiting Glioblastoma Invasion Based on Network Pharmacology and Bioinformatics
Source: Pharmaceuticals (Basel). 2024 Oct 2;17(10):1318. doi: 10.3390/ph17101318 (PMC11510114; doi:10.3390/ph17101318)
Supplement: Supplementary file 1 [file pharmaceuticals-17-01318-s001.zip › Supplemantary Figure S2.pdf]

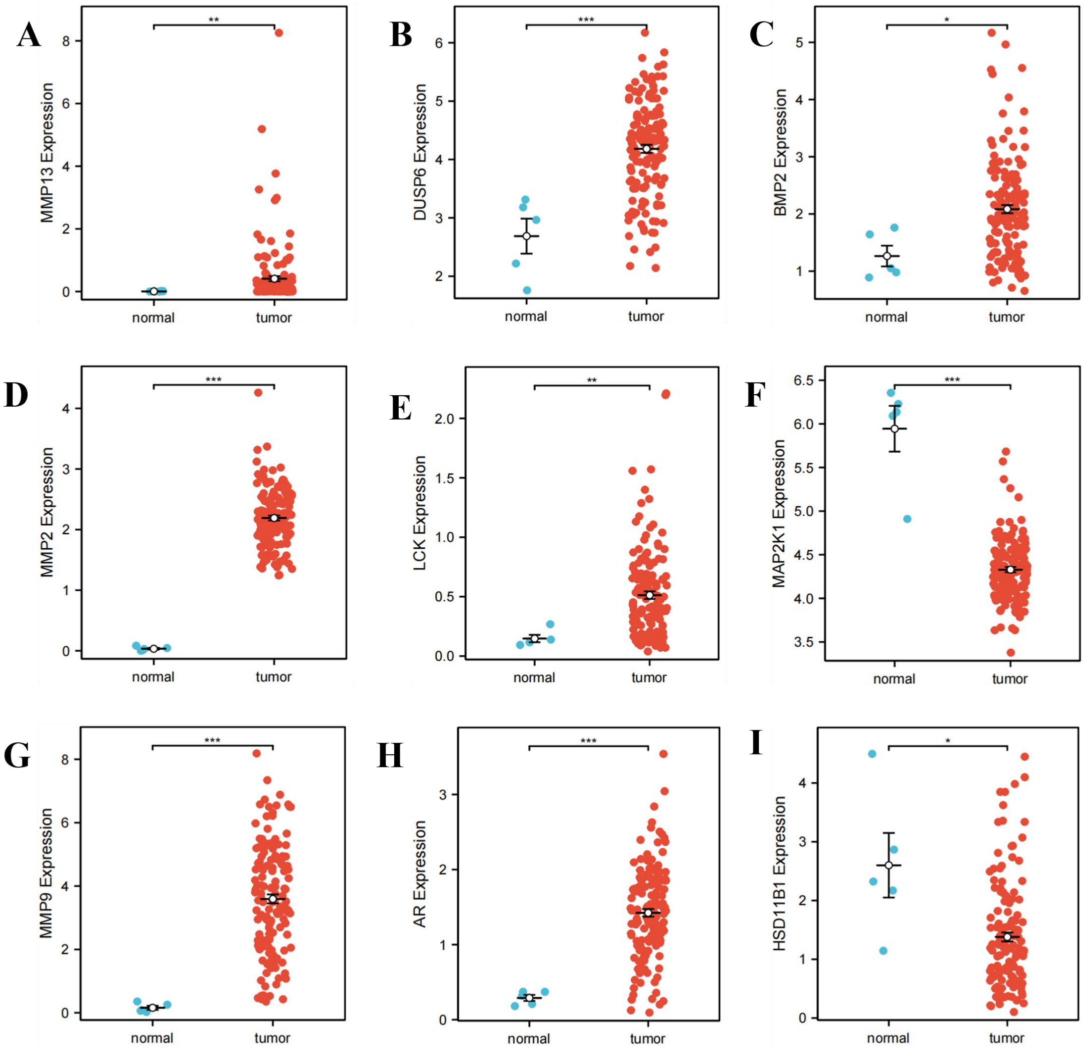

Figure S2. Differential expression analysis of nine genes between normal and tumor patients. (A-I)Box plot of *MMP13, DUSP6, BMP2, MMP2, LCK, MAP2K1, MMP9, AR,*and *HSD11B1* expression between normal and tumor patients.
