# Supplementary material for: Exploring the Mechanism of Sempervirine Inhibiting Glioblastoma Invasion Based on Network Pharmacology and Bioinformatics
Source: Pharmaceuticals (Basel). 2024 Oct 2;17(10):1318. doi: 10.3390/ph17101318 (PMC11510114; doi:10.3390/ph17101318)
Supplement: Supplementary file 1 [file pharmaceuticals-17-01318-s001.zip › Supplemantary Figure S3.pdf]

*Table S 2 . Vina Score of molecular docking about AKT1, MMP2, and MMP3 with their inhibitor*

| Compound | Targets | PDB-ID | Vina Score |
|---|---|---|---|
| MK2206 | AKT1 | 3O96 | -11.5 |
| MMP13-IN-2 | MMP2 | 7XGJ | -8.9 |
| MMP2-IN-1 | MMP13 | 4JPA | -10.0 |

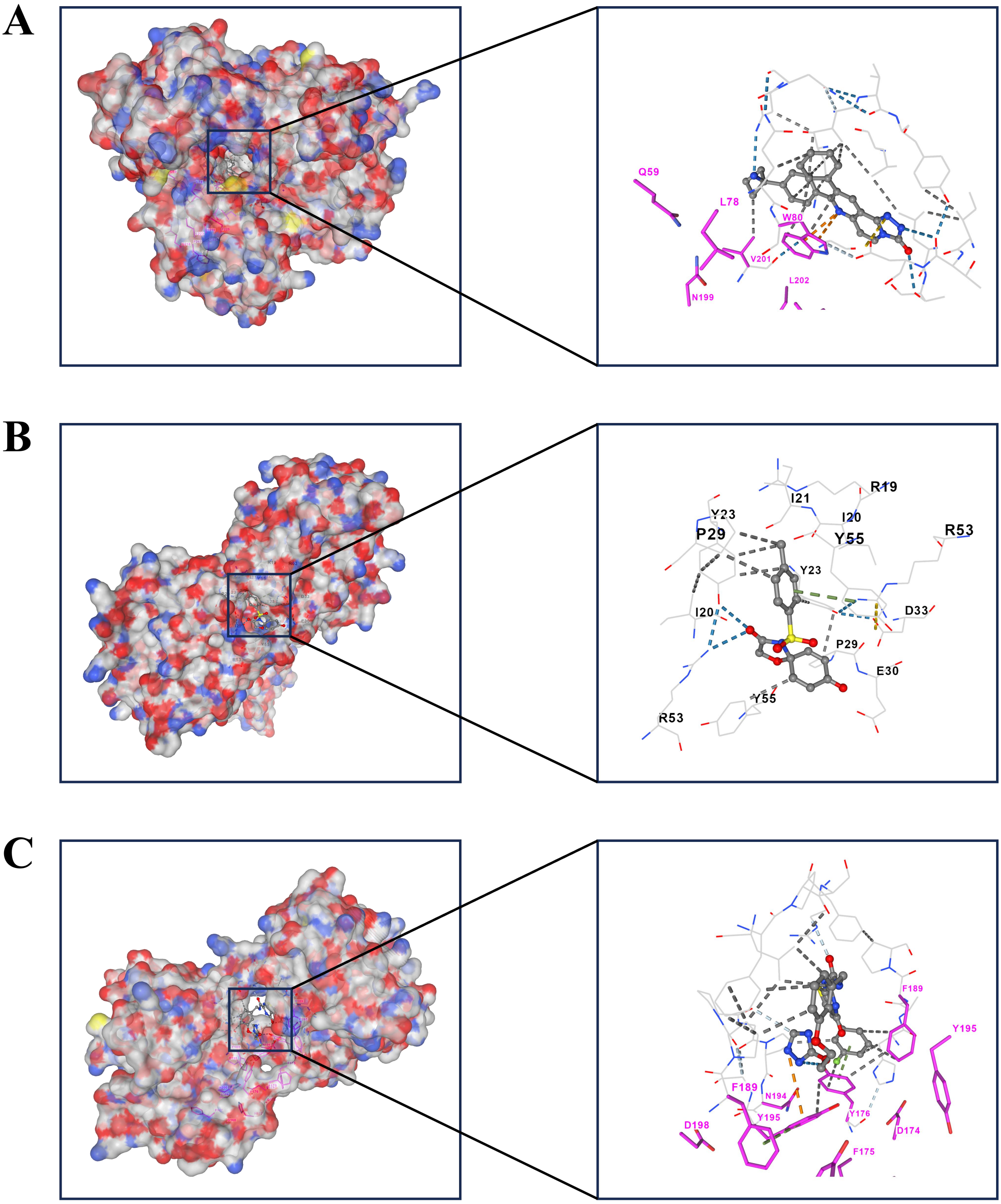


**Figure S**3. The results of molecular dockingabout AKT1, MMP2, and MMP3 with their inhibitor.(A) The docking mode of MK2206 with AKT1. (B) The docking mode of MMP2-IN-1 with MMP2. (C) The docking mode of MMP13-IN-2 with MMP13.
