# Supplementary material for: Exploring the Mechanism of Sempervirine Inhibiting Glioblastoma Invasion Based on Network Pharmacology and Bioinformatics
Source: Pharmaceuticals (Basel). 2024 Oct 2;17(10):1318. doi: 10.3390/ph17101318 (PMC11510114; doi:10.3390/ph17101318)
Supplement: Supplementary file 1 [file pharmaceuticals-17-01318-s001.zip › supplemantary Figure S4.pdf]

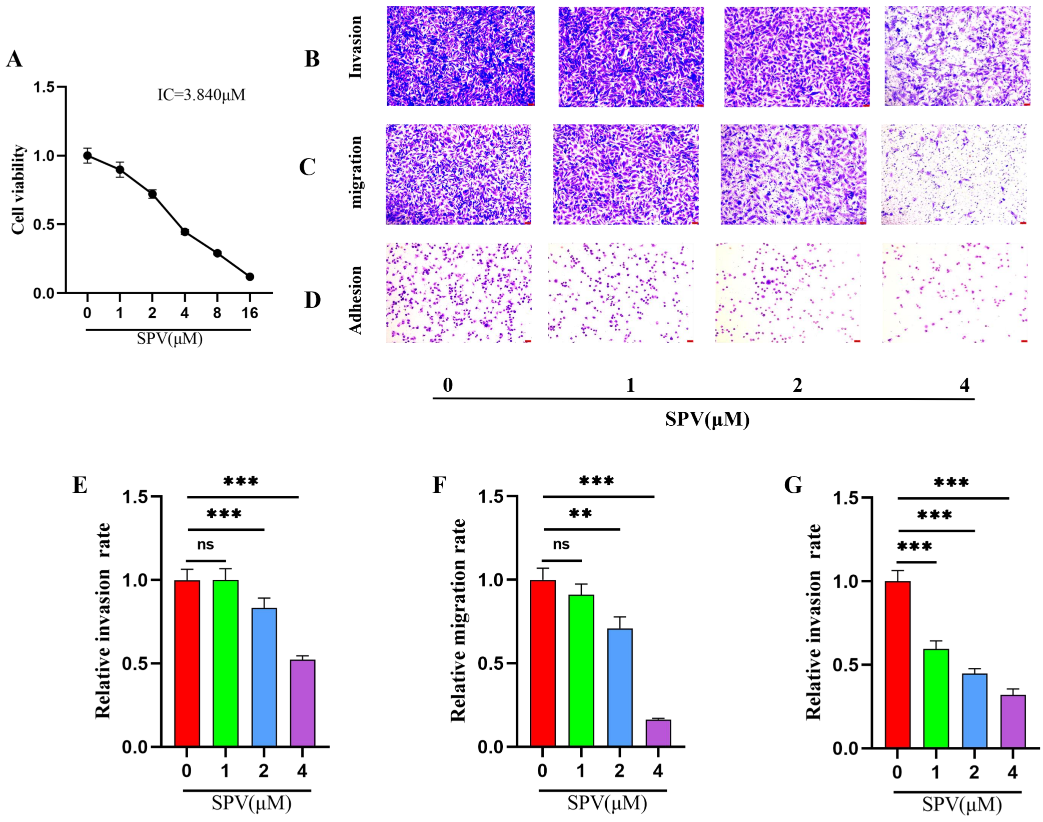

Figure S4. Sempervirine efficiently inhibits the invasion, migration and adhesion in U251 cells. (A) After SPV intervention for 48h. Cell viability was measured by CCK8 assay. (B)(E) Transwell invasion assay were treated with SPV for 36 h in U251 cells. (C)(F) Transwell migration assay of U251 cells were treated with SPV for 36 h. (D)(G) Effect of SPV on the adhesion to Matrigel coated plate after 1h exposure. The cells were stained with crystal violet to photographed(×200) and calculated by Image J software (n = 5). Data are presented as the Mean ± SD (n =5). *$p < 0.05$, **$p < 0.01$ and ***$p < 0.001$ compared with the control group.
