# Supplementary material for: Exploring the Mechanism of Sempervirine Inhibiting Glioblastoma Invasion Based on Network Pharmacology and Bioinformatics
Source: Pharmaceuticals (Basel). 2024 Oct 2;17(10):1318. doi: 10.3390/ph17101318 (PMC11510114; doi:10.3390/ph17101318)
Supplement: Supplementary file 1 [file pharmaceuticals-17-01318-s001.zip › supplemantary Figure S5.pdf]

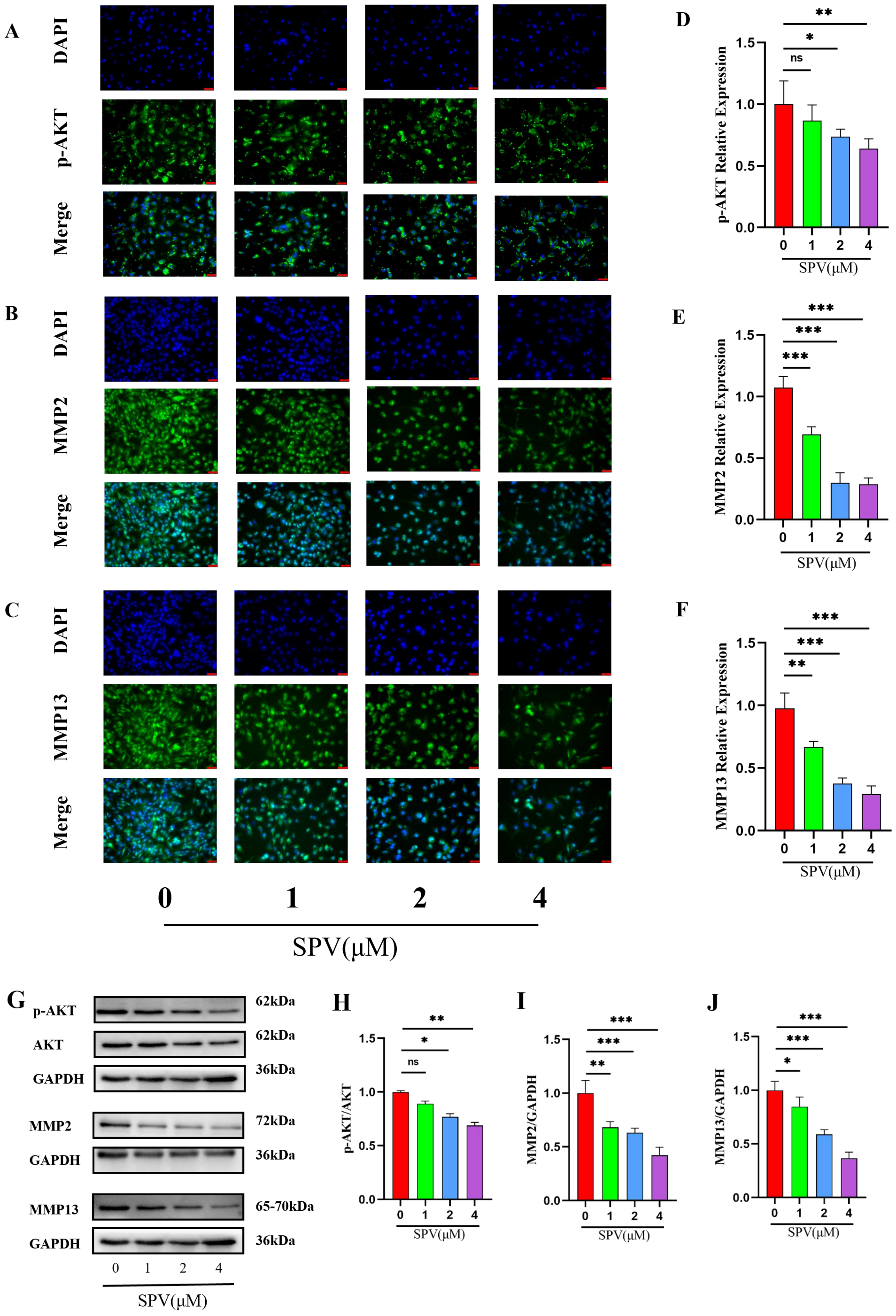

Figure S5. SPV reguletes p-AKT, MMP2 and MMP13 expression in U251 cells. (A-F) P-AKT, MMP2 and MMP13 was measured by immunofluorescence analysis of U251 cells treated with the series concentrations of SPV for 48 h. The cells were photographed(×200) and calculated by Image J software (n = 3). (G-J) P-AKT, MMP2 and MMP13 was determined by Western blotting. Data are presented as the Mean ± SD (n=3). *p < 0.05, **p < 0.01 and ***p < 0.001 compared with the control group.
