# Supplementary material for: Exploring the Mechanism of Sempervirine Inhibiting Glioblastoma Invasion Based on Network Pharmacology and Bioinformatics
Source: Pharmaceuticals (Basel). 2024 Oct 2;17(10):1318. doi: 10.3390/ph17101318 (PMC11510114; doi:10.3390/ph17101318)
Supplement: Supplementary file 1 [file pharmaceuticals-17-01318-s001.zip › Supplementary Table S1.pdf]

Table S 1 . *Information of 76 genes in TCGA samples*

Gene Symbol	Gene Name	Log2Fold Change	P-value	P-adj
GRIN1	Glutamate ionotropic receptor NMDA type subunit 1	-5.733297894	1.87814E-09	1.71086E-08
GRIN2B	glutamate ionotropic receptor NMDA type subunit 2B	-5.834467229	2.66116E-14	6.08073E-13
CHRM1	cholinergic receptor muscarinic 1	-5.029952894	1.25347E-09	1.18649E-08
MGLL	monoglyceride lipase	-5.029952894	1.25347E-09	1.18649E-08
PTGER4	prostaglandin E receptor 4	1.188045004	0.022611815	0.042371365
CYP17A1	cytochrome P450 family 17 subfamily A member 1	-2.007957017	1.70282E-05	6.76495E-05
KIF11	kinesin family member 11	2.796882304	2.04892E-13	3.9813E-12
TLR8	toll like receptor 8	2.932582888	2.72693E-06	1.28855E-05
BMP2	bone morphogenetic protein 2	1.288679287	0.014462454	0.028664386
MAPK1	mitogen-activated protein kinase 1	-1.451632973	8.17923E-12	1.22432E-10
STS	steroid sulfatase	-1.617425503	2.92651E-08	2.0936E-07
CCL24	C-C motif chemokine ligand 24	3.566874391	0.005684793	0.012554452
PIK3CG	phosphatidylinositol-4,5-bisphosphate 3-kinase catalytic subunit gamma	1.166188537	0.007515628	0.016102443
PDPK1	3-phosphoinositide dependent protein kinase 1	-1.396048789	2.16662E-12	3.57321E-11
MMP13	matrix metallopeptidase 13	6.530369619	6.14546E-06	2.6975E-05
ANXA5	annexin A5	2.730335857	9.94671E-18	4.19305E-16
MAPK8	mitogen-activated protein kinase 8	-2.746564375	2.29629E-14	5.32974E-13
KDR	kinase insert domain receptor	1.163826545	0.00047304	0.001352473
HSD11B1	hydroxysteroid 11-beta dehydrogenase 1	-1.157440575	4.13674E-07	2.31592E-06
BACE1	beta-secretase 1	-1.168833835	4.63769E-08	3.17748E-07
THRB	thyroid hormone receptor beta	-3.151233446	5.87617E-17	2.16231E-15
WAS	WASP actin nucleation promoting factor	-2.380156476	1.6941E-07	1.0304E-06
ADH5	alcohol dehydrogenase 5	1.190881328	1.69555E-09	1.56063E-08
CCNA2	cyclin A2	4.001458864	2.34003E-26	3.79192E-24
PNMT	phenylethanolamine N-methyltransferase	-1.940493425	0.021409488	0.04037302
CDK2	cyclin dependent kinase 2	3.506998154	4.74054E-46	1.19617E-42
F2	coagulation factor II	-1.795640344	0.003691619	0.008560465
CHEK1	checkpoint kinase 1	2.300150856	7.2258E-16	2.19672E-14
LCK	LCK proto-oncogene, Src family tyrosine kinase	1.943480963	0.000786097	0.002140409
TGFBR1	transforming growth factor beta receptor 1	1.323175719	2.66023E-07	1.55024E-06
EGFR	epidermal growth factor receptor	5.397601696	3.77462E-11	4.94593E-10

MDM2	MDM2 proto-oncogene	1.588541452	0.014715077	0.029089681
BCHE	butyrylcholinesterase	2.21186839	1.48703E-07	9.12625E-07
PGR	progesterone receptor	-2.51642044	7.14026E-05	0.000246904
DUSP6	dual specificity phosphatase 6	1.508623641	8.67103E-05	0.000294305
NR3C2	nuclear receptor subfamily 3 group C member 2	-1.801327911	1.45783E-05	5.88027E-05
AR	androgen receptor	2.815963138	2.043E-10	2.33866E-09
ABO	ABO	-1.068928573	0.020700403	0.039247662
ZAP70	zeta chain of T cell receptor associated protein kinase 70	1.17822295	0.003493959	0.00815565
PRKCQ	protein kinase C theta	-2.553073253	2.63318E-09	2.34307E-08
FABP6	fatty acid binding protein 6	-2.707789858	0.000117902	0.00038773
MAPKAPK2	MAPK activated protein kinase 2	1.20164753	4.76028E-10	4.99293E-09
NR1H3	nuclear receptor subfamily 1 group H member 3	1.138550397	0.000150181	0.000481686
THRA	thyroid hormone receptor alpha	-1.197688069	0.000566271	0.001592175
RBP4	retinol binding protein 4	-4.027818924	1.44196E-09	1.34901E-08
FABP7	fatty acid binding protein 7	2.448513327	5.6106E-05	0.000198359
ITGAL	integrin subunit alpha L	1.350276336	0.001880892	0.004676549
ESRRG	estrogen related receptor gamma	-1.105390742	0.00174455	0.00437016
SYK	spleen associated tyrosine kinase	1.465779845	0.000172507	0.000545664
ERBB4	erb-b2 receptor tyrosine kinase 4	-2.330044754	2.73939E-05	0.000104124
DPP4	dipeptidyl peptidase 4	2.74244203	0.000174044	0.000549544
NR1I3	nuclear receptor subfamily 1 group I member 3	-1.374734496	9.23079E-08	5.8903E-07
HMGCR	3-hydroxy-3-methylglutaryl-CoA reductase	-1.314455825	5.57129E-07	3.02494E-06
BRAF	B-Raf proto-oncogene, serine/threonine kinase	-1.431365578	2.0203E-14	4.75794E-13
CASP3	caspase 3	1.60506252	1.01224E-08	7.99249E-08
FABP3	fatty acid binding protein 3	-2.426273608	1.13727E-08	8.91199E-08
GSTP1	glutathione S-transferase pi 1	1.204607701	1.89479E-06	9.24264E-06
MAP2K1	mitogen-activated protein kinase kinase 1	-1.954204551	5.71866E-25	7.89131E-23
MMP2	matrix metallopeptidase 2	3.893396056	3.12707E-15	8.45841E-14
TEK	TEK receptor tyrosine kinase	3.51833603	8.07186E-05	0.000276198
HCK	HCK proto-oncogene, Src family tyrosine kinase	1.593131055	0.000239426	0.000732163
F7	coagulation factor VII	-2.338622541	0.007702345	0.016458567
SERPINA1	serpin family A member 1	2.777334646	1.23597E-07	7.72229E-07
PLA2G2A	phospholipase A2 group IIA	6.710616647	5.07241E-10	5.2889E-09
MMP8	matrix metallopeptidase 8	6.64382632	7.90549E-06	3.3818E-05
HMOX1	heme oxygenase 1	2.651661988	9.32346E-07	4.85496E-06
S100A9	S100 calcium binding protein A9	3.381781511	1.73E-06	8.51E-06

CTSK	cathepsin K	2.453712287	1.33943E-05	5.45122E-05
KIT	KIT proto-oncogene, receptor tyrosine kinase	-1.409633241	0.024237828	0.044946222
CASP1	caspase 1	2.951421368	9.96495E-12	1.46554E-10
TGM3	transglutaminase 3	-1.422216329	0.012018599	0.02435014
DPEP1	dipeptidase 1	6.821974431	3.00E-20	1.90E-18
MMP9	matrix metallopeptidase 9	7.321419017	1.89943E-20	1.24258E-18
PROCR	protein C receptor	2.446579602	6.38516E-10	6.47875E-09
PIK3R1	phosphoinositide-3-kinase regulatory subunit 1	-1.032315636	0.002008943	0.004953093
TGFB2	transforming growth factor beta 2	1.296632366	0.00670579	0.014550906
